# Associations between Gender Expression, Protective Coping Strategies, Alcohol Saliency, and High-Risk Alcohol Use in Post-Secondary Students at Two Canadian Universities

**DOI:** 10.3390/ijerph21010107

**Published:** 2024-01-18

**Authors:** Anees Bahji, Paul Boonmak, Michelle Koller, Christina Milani, Cate Sutherland, Salinda Horgan, Shu-Ping Chen, Scott Patten, Heather Stuart

**Affiliations:** 1 Department of Psychiatry, Cumming School of Medicine, University of Calgary, Calgary, AB T2N 4N1, Canada; patten@ucalgary.ca; 2 Department of Community Health Sciences, Cumming School of Medicine, University of Calgary, Calgary, AB T2N 4N1, Canada; 3 Department of Public Health Sciences, Queen’s University, Kingston, ON K7L 3N6, Canada; 4 School of Rehabilitation Therapy, Queen’s University, Kingston, ON K7L 3N6, Canada; 5 Department of Occupational Therapy, Faculty of Rehabilitation Medicine, University of Alberta, Edmonton, AB T6G 2R3, Canada

**Keywords:** gender expression, high-risk alcohol use, protective coping strategies, Canadian universities

## Abstract

Background: This study, conducted in October 2017 at two Canadian universities, aimed to explore the relationships between gender expression, protective coping strategies, alcohol saliency, and high-risk alcohol use. Methods: Validated scales were employed to assess these variables using survey data. Multivariate analyses were conducted to investigate the associations between these factors and high-risk drinking. Results: This study revealed significant associations between high-risk drinking and androgynous gender roles (OR = 1.58, 95% CI: 1.19–2.10) as well as among self-reported males (OR = 2.21; 95% CI: 1.77–2.75). Additionally, protective behavioural strategies were inversely related to high-risk drinking (OR = 0.95; 95% CI: 0.94–0.96), while higher alcohol saliency exhibited a positive correlation with high-risk drinking (OR = 1.12; 95% CI: 1.11–1.14). Conclusions: These findings underscore the importance of considering gender, alcohol saliency beliefs, and protective behavioural strategies in the development and refinement of interventions aimed at reducing high-risk alcohol use on Canadian campuses.

## 1. Introduction

Excessive alcohol consumption is a longstanding, significant public health issue experienced worldwide [1,2]. In Canada, data from the 2019–2020 Canadian Postsecondary Education Alcohol and Drug Use Survey (CPEADS) revealed that approximately 60% of individuals who consumed alcohol in the past 30 days engaged in heavy drinking, 74% of those who drank in the last month reported feeling intoxicated at least once, and that approximately 56% of those who drank in the past year encountered at least one negative alcohol-related consequence [3]. Heavy alcohol consumption among post-secondary students can lead to adverse sequelae, including poor mental health, cognitive problems, violence, academic underperformance, financial strain, and gastrointestinal issues [4]. Multiple factors affect hazardous college alcohol consumption, including sex, genetic susceptibility, campus alcohol norms, expectations, penalties for underage drinking, and alcohol availability [5]. Although post-secondary institutions have implemented various interventions to address some or all of these factors [6,7], alcohol-related problems persist among college students worldwide. This suggests a further need to determine the association between alcohol use and other risk factors among Canadian post-secondary students. This can help guide better preventative interventions to improve student health and wellbeing on Canadian campuses.

Despite the importance of biological, psychological, and social variables, and the distinctions of sex and gender, few studies have examined the role of gender in alcohol consumption among post-secondary students [8]. Sex refers to the innate biological differences between males and females, and gender refers to the expression of sex and the accompanying social roles an individual adopts [9,10,11,12]. Using biological models, male post-secondary students have consistently consumed more alcohol and experienced more alcohol-related harms than their female counterparts, even after accounting for age, education, familial risk, and concurrent mental health difficulties [13,14,15]. However, less is known about the role of gender identity (i.e., one’s core sense of self as a man, woman, or neither) and gender expression (i.e., the gender-related stereotypes one fulfills). The two concepts of gender role and expression differ in that expectations of gender roles result from societal assumptions about the biological differences between men and women [9,16]. Gender disparities in alcohol use behaviours may come from differing gender conformity, orientation, and expression pressures [17]. Traditionally, masculine traits (e.g., independent, assertive, leadership traits) have been positively associated with high-risk drinking independent of biological sex. At the same time, those with feminine traits (e.g., compassion, understanding, sympathy) appear to be at a decreased risk for alcohol-related harm [18,19,20]. As modern mental health programs increasingly align to gender rather than sex (e.g., a transgender woman would be treated as a woman), gender-based alcohol research in the extant literature is a priority area.

Previous research has demonstrated that beliefs about the role of alcohol in college life, termed alcohol salience beliefs, mediate the degree of alcohol-related harm among post-secondary students [21,22,23,24]. College students who believe alcohol is central to college life and campus culture are more prone to hazardous alcohol consumption [22,23]. Angosta et al. furthered this work by demonstrating how self-identification with other college students was a significant moderator of the association between alcohol salience beliefs, frequency of drinking, and the peak number of drinks [21]. Most of this work has been conducted using a biological sex model, where alcohol salience beliefs are more strongly associated with higher drinking among biological males than females. However, little is known about how alcohol salience relates to both gender identity and gender expression.

Related to alcohol salience beliefs, protective behavioural strategies (PBSs) are specific behaviours one can utilize to minimize the harmful consequences of alcohol consumption, including injuries, assaults, and death [25,26]. Examples of PBSs include alternating between alcoholic and non-alcoholic drinks, planning limits on drinking, drinking slowly, using a designated driver, and knowing where your drink has been [25,26]. Previous studies have examined the moderating effects of sex on the relationship between PBS use and alcohol problems and indicate that PBSs are more protective for women than men against experiencing alcohol-related problems [22,27]. More traditionally feminine gender expression seems to predict greater use of protective behavioural strategies [27,28,29,30], which reduce the negative consequences of alcohol consumption [31]. Previous studies suggest conformity to male gender norms moderates the relationship between PBSs and hazardous alcohol use [27,29,32]. However, these studies have examined gender trends among predominantly biological male samples; hence, little is known about how PBSs relate to gender identity and gender expression among more diverse samples concerning alcohol use.

This study determined the frequency and associations between hazardous alcohol use, gender, alcohol salience behaviours, and protective behavioural strategies in two large metropolitan Canadian universities. Due to the limited literature in this area, these results may help to shape preventative interventions. We hypothesized that alcohol misuse would be associated with masculine gender expression and identity, lower protective behavioural strategies, and greater alcohol salience.

## 2. Materials and Methods

### 2.1. Study Design, Procedure, and Sample

The current study was part of a larger research initiative to raise awareness of the associations between gender, mental health, and high-risk alcohol behaviours among Canadian post-secondary students. For the present study, we utilized data from an online cross-sectional survey administered to a sample of university students in October 2017. The survey participants were enrolled at two Canadian universities whose names have been withheld for confidentiality. Ethics approval for this study was obtained from the Research Ethics Boards at both universities. A survey link was sent to each university’s undergraduate students to recruit participants. There were no specific eligibility criteria apart from the need to be enrolled as an undergraduate at either university. Once students agreed to participate, they were directed to complete the online questionnaire. To maximize participation, a reminder email was sent out. Three thousand four hundred forty-six students (32.9% response rate) finished the questionnaire across two universities. Among them, 2226 students were from one university (representing 9% of the overall undergraduate population of 24,143 students), while 1220 students were from the other university (representing 4% of the overall undergraduate population of 30,000 students).

### 2.2. Measures

The questionnaire collected data on demographics, levels of alcohol consumption, gender identity, gender expression, protective behavioural strategies, and alcohol salience beliefs. The demographic data collected included age, university attended, and the year of study (i.e., the number of years a student has been enrolled).

#### 2.2.1. Alcohol Use

To assess levels of alcohol consumption, the Alcohol Use Disorders Identification Test (AUDIT) was used [33]. The AUDIT is a quick, self-administrated screening tool to evaluate harmful alcohol use. It is a 10-item questionnaire scored from 0 to 4 points for each item with an overall range of 0 to 40 [34]. Previous research has shown that AUDIT is reliable and valid for detecting alcohol dependence among college students [35,36,37]. As per previous post-secondary validation studies [35,36,37], we used a binary interpretation using a total score of 7 for males and 5 for females; above these scores, individuals were categorized as having high-risk alcohol use.

#### 2.2.2. Gender Identify and Gender Expression

We included two gender measures. We used dichotomous gender self-categorization (e.g., masculine or feminine) to measure gender identity and used the Bem Gender Role Inventory (BGRI) to determine gender expression [38]. The BGRI is a self-administered screening tool that determines the alignment of personality characteristics with four traditional gender roles: masculine, feminine, androgynous, or undifferentiated [38]. It is a 12-item questionnaire scored from 0 to 5 points for each item with an overall range of 0 to 60 [38]. The BGRI has six stereotypically masculine attributes (e.g., leadership abilities, strong personality, dominant, defends beliefs) and six traditionally feminine attributes (e.g., warm, gentle, affectionate, sympathetic). All respondents have a “masculine” and “feminine” score; the assignment to the gender roles overlaps with the relative scoring on the masculine and feminine scales. For example, the “masculine” identity refers to scoring high on the masculine scale and low on the feminine scale. In contrast, scoring high on both masculine and feminine scales results in the “androgynous” gender role; scoring low on both scales results in the “undifferentiated” gender role. Finally, it is important to note that the 12-item BGRI is a valid tool for post-secondary students [39].

#### 2.2.3. Beliefs about Alcohol Use 

The College Life Alcohol Screening Scale (CLASS) was used to determine respondents’ internalization of alcohol’s role in campus culture [40]. The CLASS measures beliefs about alcohol’s centrality to the college experience and drinking culture [21,41]. It is a 15-item questionnaire scored from 0 to 5 points for each item, with an overall range of 0 to 75. The CLASS was scored by summing the scores on all 15 items after reverse-scoring items 3 and 8 [21,41]. Higher CLASS scores indicate greater alcohol salience.

#### 2.2.4. Protective Behavioural Strategies

The Protective Behavioural Strategies Scale (PBSS) was utilized to determine the degree of protective behavioural strategies [41,42]. PBSS is a self-administered screening tool that determines how respondents use coping skills to prevent alcohol-related harms. The PBSS is a 15-item questionnaire scored from 0 to 5 points for each item, with an overall range of 0 to 75 [43]. The PBSS considers three categories of protective behavioural strategies: Stopping/Limiting Drinking (e.g., predetermining a set number of drinks not to exceed, leaving the bar/party at a predetermined time), Manner of Drinking (e.g., avoiding drinking games, drinking slowly, avoiding mixing types of alcohol), and Serious Harm Reduction (e.g., using a designated driver, knowing where your drink has been at all times) [41,44]. Higher overall PBSS scores indicate greater utilization of protective behavioural strategies. 

### 2.3. Data Analysis

We used the *survey* package in *R* (version 4.1.) for all analyses, utilized within the R-Studio interface (version 3.5.3, Boston, MA, USA) [45,46]. First, we summarized the overall and university-stratified participant demographics using descriptive statistics using built-in statistical packages within *R* (Table 1). We used chi-square tests and *t*-tests to compare differences between categorical and continuous variables; *p*-values less than 0.05 were considered statistically significant. We also calculated 95% confidence intervals using the Agresti–Coull method to compare the frequency of high-risk versus low-risk drinking across each BGRI category. Second, we calculated Pearson’s correlation coefficients to measure the association between scores on the AUDIT, the PBSS, and the CLASS. Third, we constructed individual binary logistic regression models using the *syvglm* function within the *survey* package to specify generalized linear models (GLMs). For each model, we examined the association between predictor variables (gender identity, gender expression, year of study, beliefs about alcohol, coping skills) with dichotomized AUDIT score as a dependent variable (Table 2). We estimated the odds ratio (OR) and the 95% confidence interval. Finally, using *svyglm*, we developed a multivariable logistic regression model including all variables used in the bivariate logistic regression models (Table 3). We generated adjusted odds ratios (ORs) and their associated 95% confidence intervals in these models. Next, we calculated the point-biserial correlation coefficient (r-value) between self-reported gender identity and the BGRI, which was <0.001. As this indicates a weak correlation between the two variables, the multicollinearity issue arising from including both variables in the same model was felt negligible. Therefore, we included self-reported gender identity and the BGRI in the same multivariable model.

## 3. Results

### 3.1. Demographics

A total of 3466 participants aged 18–25 (M = 19.72, SD = 1.63) completed the survey. Across the sample, most participants self-identified as female (71.1%, *n* = 2027). Table 1 describes the sampling distribution in terms of age, year of study, BGRI, self-reported gender identity, and high-risk alcohol use, stratified by the university. Regarding the year of study, approximately a third of the sample (33%) was in their first year, while 15%, 23%, 22%, and 7% were in their second through fifth years, respectively. The most prevalent BGRI across universities was androgynous (35%), followed by feminine (25%), masculine (22%), and undifferentiated (17%).

### 3.2. Frequency of Alcohol Use

The results for the AUDIT score ranged from 1 to 35 (M = 7.61, SD = 4.74), and males (M = 9.04, SD = 5.32) had a slightly higher average score than females (M = 7.02, SD = 4.49; t = 10.30; *p*-value < 0.01). By BGRI across universities, the frequency of high- versus low-risk drinking (alongside their 95% Agresti–Coull confidence intervals) was highest among those with an androgynous BGRI (16.9% [15.2–18.8%] vs. 18.1% [16.3–20.1%]), followed by masculine (11.8% [10.3–13.5%] vs. 10.3% [8.9–11.8%]), feminine (10.7% [9.3–12.3%] vs. 14.6% [13.0–16.4%]), and undifferentiated (7.2% [6.0–8.6%] vs. 10.3% [8.9–11.9%]). However, only among those with a masculine BGRI was the frequency of high-risk drinking higher than that of low-risk drinking. By self-reported gender identity, the frequency of high- versus low-risk drinking (alongside their 95% Agresti–Coull confidence intervals) was higher among males (33.9% [31.0–36.9%] vs. 18.6% [16.3–21.0%]) than females (11.5% [10.4–12.7%] vs. 33.0% [31.5–34.6%]; *p* < 0.01). 

### 3.3. Bivariate Statistics

The bivariate correlations (measured using the Pearson correlation coefficient) between CLASS and AUDIT (r = 0.58), PBSS and AUDIT (r = −0.46), and CLASS and PBSS (r = −0.45) indicated moderate positive correlations between alcohol saliency and high-risk drinking and moderate negative correlations between protective behavioural strategies and both alcohol salience and high-risk drinking. By BGRI categories across universities, the mean (SD) total CLASS scores were highest for masculine individuals (41.3 ± 11.1), androgynous (38.8 ± 11.2), undifferentiated (38.3 ± 11.1), and lowest for feminine (37.5 ± 10.4). By self-reported gender across universities, CLASS scores were higher among males (42.1 ± 9.8) than females (35.6 ± 10.2). Across universities, the mean (SD) total PBSS scores were highest for androgynous (69.6 ± 11.4), followed by feminine (69.1 ± 10.3), androgynous (65.2 ± 10.6), and masculine Bem gender expression (64.9 ± 11.8). By self-reported gender across universities, PBSS scores were higher among females (72.4 ± 11.2) than males (62.8 ± 7.7).

### 3.4. Regression Analyses

Table 2 presents the findings from simple unadjusted logistic regression models investigating the relationship between various factors and high-risk drinking, as measured using the AUDIT risk category. Unadjusted models revealed no significant association between year of study, age, and self-reported gender identity with high-risk drinking. However, individuals with masculine (OR = 1.64; 95% CI: 1.32–2.05) or androgynous gender roles (OR = 1.34, 95% CI: 1.10–1.64), compared to those with an undifferentiated Bem gender role, exhibited higher odds of engaging in high-risk drinking. Conversely, feminine gender roles did not show such an association in bivariate analyses. Moreover, increased scores on protective behavioural strategies (considered a continuous variable) were associated with reduced problem drinking (OR = 0.93; 95% CI: 0.92–0.94). Upon examining the individual subscales, the MOD score (OR = 0.77; 95% CI, 0.75–0.79), SHR (OR = 0.89; 95% CI, 0.86–0.91), and the SLD (OR = 0.95; 95% CI, 0.93–0.96) each demonstrated protective effects against meeting the criteria for high-risk drinking. Lastly, higher alcohol salience (measured by increased scores on the CLASS) was associated with higher odds of problem drinking (OR = 1.16; 95% CI: 1.15–1.17).

### 3.5. Multivariable Regression Results

Table 3 shows the results of multivariable logistic regression models examining the association between all primary variables. Greater protective behavioural strategies scale scores remained associated with reduced problem drinking (OR = 0.95; 95% CI: 0.94–0.96), while higher CLASS scores were still associated with greater problem drinking (OR = 1.12; 95% CI: 1.11–1.14). Those who adhered to an androgynous gender role (OR = 1.58, 95% CI: 1.19–2.10) were likelier to engage in problem drinking. However, the masculine and feminine BGRI categories were no longer associated with drinking. However, self-reported gender identity as male was associated with more than double the odds of high-risk drinking than female status (OR = 2.21; 95% CI, 1.77–2.75).

## 4. Discussion

The present study explores the relationships between alcohol use, gender, alcohol salience, and protective behavioural strategies among post-secondary students at two Canadian universities. Notably, most participants reported answers consistent with the androgynous BGRI. Across the sample, higher utilization of protective behavioural strategies and lower alcohol saliency was found to have a protective effect against the high-risk use of alcohol. Among participants, the frequency of high-risk drinking was highest among those with an androgynous identity, followed by masculine, feminine, and undifferentiated BGRI categories. We also found that only individuals endorsing a masculine BGRI exhibited a higher frequency of high-risk drinking than low-risk drinking. These findings correspond with the categorization of high-risk drinking based on self-reported gender identity (male versus female), wherein the frequency of high-risk drinking was significantly greater among males (33.9% vs. 18.6%) than females (11.5% vs. 33.0%, *p* < 0.001).

In the present analyses, we included self-reported gender identity and gender expression (measured using the BGRI) in the multivariable models, allowing us to examine differences based on self-identification. Only the androgynous BGRI remained significantly associated with high-risk drinking, even after adjusting for self-reported gender identity, alcohol salience, and protective strategies. Ultimately, this finding indicates that gender expression, as measured using the BGRI, plays a distinct role in alcohol use, separate from self-reported gender identity. Furthermore, this finding is consistent with the existing literature addressing the differentiation between biological sex and gender identity in cisgender young adults. Although gender identity is a multifaceted construct influenced by biological, social, and environmental factors, the precise mechanisms underlying its development are still not fully understood. Some studies have suggested the presence of brain differences between individuals with different gender identities. For instance, research utilizing brain imaging techniques such as MRI scans has revealed variations between cisgender and transgender individuals [47].

Alcohol salience beliefs greatly affected the prediction of high-risk AUDIT scores on both surveyed campuses. Previous research by Angosta and colleagues demonstrated that self-identification with other students was the most significant moderator between alcohol salience and alcohol consumption [21]. Hence, alcohol salience beliefs are a worthwhile target for reducing high-risk alcohol use on campus [24]. Consequently, it may be important to shift students’ perceptions of drinking as an important part of the college experience, particularly for those who identify with their peers [21].

As per the Canadian Centre on Substance Use and Addiction and National Institutes on Alcohol Abuse and Alcoholism, effective strategies for reducing post-secondary alcohol-related harms involve a combination of peer-to-peer education, training in the use of protective coping strategies, and skills to manage social pressures to drink can reduce the harms of heavy drinking on campus [48]. To that end, individual-level interventions, such as brief motivational interviewing, reduce alcohol use in post-secondary students and lower the incidence of alcohol-related problems over longer follow-up intervals [49]. Individual-level interventions can target high-risk behaviours (e.g., avoiding “pregaming” [50]) or high-risk groups (e.g., first-year, mandated, or minority students) [51,52].

In parallel, environmental-level strategies target the campus community and student body. These are designed to change the campus and community environments where student drinking occurs (e.g., reducing alcohol availability [53,54], and community liaison and security services to reduce alcohol consumption and alcohol-related aggression [55]). However, most of the available studies have pointed to a need to explore gender and social moderators for the efficacy of these interventions.

Our findings emphasize the importance of effective screening, identification, and interventions to address high-risk drinking among Canadian post-secondary students. Targeting the entire student population is crucial and can be achieved by incorporating screening to ensure effective screening. Measures as part of routine health assessments or via online platforms allow students to complete self-assessment questionnaires privately and confidentially. Collaborating across campus agencies, including health services, counselling, and student organizations, can also help facilitate screening tools to identify high-risk users of alcohol and raise awareness of the importance of early interventions.

Furthermore, specific psychosocial interventions can help build practical coping skills in post-secondary students to reduce alcohol use. Several approaches have shown promise, including brief motivational interviewing, which includes a short one-on-one session with a trained counsellor to explore and address an individual’s motivations, values, and goals around alcohol use. Brief motivational interviewing has been found to reduce alcohol consumption and related problems among college students effectively [56,57]. Another approach is cognitive-behavioural therapy (CBT), which focuses on identifying and modifying maladaptive thoughts and behaviours associated with alcohol use. CBT has reduced heavy drinking and associated negative consequences among college students in both group and individual settings [58].

### Strengths and Limitations

Our study has several limitations that warrant consideration. We employed a convenience sampling method, which may limit the generalizability of our findings. The distribution of self-reported gender identities in our sample differs from the broader university population, raising concerns about potential biases when comparing our convenience sample to the entire student body. Variations in gender representation across universities also underscore the limitations of this approach. Additionally, we assessed gender identity using a binary choice, which fails to capture the nuanced experiences of individuals with diverse gender identities, such as non-binary individuals, potentially affecting the comprehensiveness of our findings. The categorization of alcohol use severity based on AUDIT scores may introduce the potential for misclassification errors, as it may not fully capture the complexity of alcohol consumption patterns and their consequences. Moreover, the historical nature of the BGRI measure employed in our study, originating from the 1970s [59], may not fully align with contemporary understandings of gender roles and identities [60], although it remains commonly used in gender-related research [61]. Our survey did not collect data on other potential risk factors for high-risk alcohol use, such as family history, mental illness, or concurrent substance use, limiting the comprehensiveness of our analysis. Furthermore, the incentivization for participation in our study may have introduced some participation bias, as individuals with a specific interest or motivation to complete the survey may differ from the broader student population. Lastly, although the rate of missing data in our study was minimal (<1%), the exclusion of these cases carries a potential risk of selection bias and may have affected certain outcome measures. Despite these limitations, our study’s strengths, including a large sample size and a unique focus on gender rather than solely biological sex, contribute valuable insights to the field. It is essential to interpret our findings within the context of these limitations and recognize the need for future research to address these issues more comprehensively.

## 5. Conclusions

In this cross-sectional study, higher protective behavioural strategy and lower alcohol salience belief scores were associated with lower odds of high-risk alcohol use. These may be useful targets for intervention. These findings provide a rationale for incorporating gender, alcohol salience beliefs, and protective behavioural strategies into developing and refining intervention strategies for reducing high-risk alcohol use on Canadian campuses. Given the differences between the two schools, our results suggest the need for campus-specific interventions and local needs assessments.

## Figures and Tables

**Table 1:** Basic demographics of study sample by age and year of study for each university.

	University 1	University 2	Chisq (*p*)	Total
Total	2226 (100.0%)	1220 (100.0%)		
Year of Study			χ^2^_(4)_ = 101.76, *p* < 0.01	3446 (100.0%)
1st year	828 (37.2%)	319 (26.1%)		1147 (33.3%)
2nd year	304 (13.7%)	221 (18.1%)		525 (15.2%)
3rd year	509 (22.9%)	272 (22.3%)		781 (22.7%)
4th year	486 (21.9)	264 (21.6%)		750 (21.8%)
5th year	97 (4.4%)	144 (11.8%)		241 (7.0%)
Gender Identity			χ^2^_(2)_ = 117.47, *p* < 0.01	
Male	588 (26.4%)	235 (19.3%)		823 (12.2%)
Female	1366 (61.4%)	661 (54.2%)		2027 (19.3%)
Missing	272 (12.2%)	324 (26.7%)		596 (54.2%)
Bem Gender Role			χ^2^_(4)_ = 45.83, *p* < 0.01	
Masculine	518 (23.8%)	229 (19.1%)		747 (22.1%)
Feminine	509 (23.4%)	348 (29.0%)		857 (25.4%)
Androgynous	818 (37.6%)	365 (30.6%)		1183 (35.1%)
Undifferentiated	332 (14.9%)	256 (21.4%)		588 (17.4%)
Missing	49 (2.2%)	22 (1.8%)		71 (2.0%)
Alcohol Use			χ^2^_(2)_ = 255.24, *p* < 0.01	
Low Risk	942 (43.4%)	834 (71.8%)		1776 (53.3%)
High Risk	1228 (56.6%)	328 (28.2%)		1556 (46.7%)
Missing	56 (2.5%)	58 (4.8%)		114 (4.8%)
AUDIT Total	Mean: 8.5, SD: 4.9, 95% CI: (8.3, 8.7)	Mean: 5.7, SD: 3.7, 95% CI: (5.5, 5.9)		Mean: 7.6, SD: 4.7, 95% CI: (7.4, 7.8)
CLASS Total	Mean: 41.4, SD: 10.5, 95% CI: (41.0, 41.8)	Mean: 34.6, SD: 5.8, 95% CI: (34.3, 34.9)		Mean: 39.0, SD: 11.1, 95% CI: (38.6, 39.4)
PBSS Total	Mean: 66.7, SD: 11.4; 95% CI: (66.2, 67.2)	Mean: 69.7, SD: 10.9, 95% CI: (69.1, 70.3)		Mean: 67.6, SD: 11.3, 95% CI: (67.2, 68.0)

AUDIT = Alcohol Use Disorders Identification Test; PBSS = Protective Behavioural Strategies Scale; CLASS = College Life Alcohol Salience Scale; SD = Standard Deviation; CI = Confidence Interval; (χ) is the degree of freedom for the test. For example, in the Year of Study, there are 5 options (1, 2, 3, 4, 5), so there are n-1 (4) degrees of freedom for the chisquare test.

**Table 2:** Relationships between demographic variables, Bem gender roles, protective behavioural strategies, and alcohol salience beliefs to high-risk alcohol use.

Variable	OR for High-Risk AUDIT Score
Year of Study (per year)	1.05 (0.99–1.10)
Per Year Older	0.99 (0.95–1.03)
Gender Identity (Male vs. Female)	1.02 (0.86–1.20)
Masculine Gender Role (vs. Undifferentiated)	1.64 (1.32–2.05) *
Androgynous Gender Role (vs. Undifferentiated)	1.34 (1.10–1.64) *
Feminine Gender Role (vs. Undifferentiated)	1.06 (0.85–1.31)
PBSS Total Score (per point increase)	0.93 (0.92–0.94) *
MOD Subscore (per point increase)	0.77 (0.75–0.79) *
SHR Subscore (per point increase)	0.89 (0.86–0.91) *
SLD Subscore (per point increase)	0.95 (0.93–0.96) *
CLASS Total Score (per point increase)	1.16 (1.15–1.17) *

OR = odds ratio; AUDIT = Alcohol Use Disorders Identification Test; PBSS = Protective Behavioural Strategies Scale; CLASS = College Life Alcohol Salience Scale. * = statistically significant.

**Table 3:** Multivariable logistic regression model for relationships between Bem gender roles, protective behavioural strategies, and alcohol salience to high-risk drinking.

Variable	OR for High-Risk AUDIT Score
University	0.50 (0.41–0.61)
Year of Study (per year)	0.99 (0.93–1.07)
CLASS Total Score (per point increase)	1.12 (1.11–1.14)
PBSS Total Score (per point increase)	0.95 (0.94–0.96)
Androgynous Gender Role (vs. Undifferentiated)	1.58 (1.19–2.10)
Masculine Gender Role (vs. Undifferentiated)	1.26 (0.94–1.71)
Feminine Gender Role (vs. Undifferentiated)	1.23 (0.92–1.65)
Gender Identity (Male vs. Female)	2.21 (1.77–2.75)

OR = odds ratio; AUDIT = Alcohol Use Disorders Identification Test; PBSS = Protective Behavioural Strategies Scale; CLASS = College Life Alcohol Salience Scale.

## Data Availability

Restrictions apply to the availability of these data. Data were obtained from the Caring Campus Survey and are not available for release due to privacy and confidentiality concerns.

## References

[1] Degenhardt L., Charlson F., Ferrari A., Santomauro D., Erskine H., Mantilla-Herrara A., Whiteford H., Leung J., Naghavi M., Griswold M. (2018). The Global Burden of Disease Attributable to Alcohol and Drug Use in 195 Countries and Territories, 1990–2016: A Systematic Analysis for the Global Burden of Disease Study 2016. Lancet Psychiatry.

[2] Griswold M.G., Fullman N., Hawley C., Arian N., Zimsen S.R.M., Tymeson H.D., Venkateswaran V., Tapp A.D., Forouzanfar M.H., Salama J.S. (2018). Alcohol Use and Burden for 195 Countries and Territories, 1990–2016: A Systematic Analysis for the Global Burden of Disease Study 2016. Lancet.

[3] Canadian Centre on Substance Use and Addiction Canadian Postsecondary Education Alcohol and Drug Use Survey. 2019–2020. https://pepah.ca/cpads-toolkit/.

[4] Tembo C., Burns S., Kalembo F. (2017). The Association between Levels of Alcohol Consumption and Mental Health Problems and Academic Performance among Young University Students. PLoS ONE.

[5] White A., Hingson R. (2013). The Burden of Alcohol Use: Excessive Alcohol Consumption and Related Consequences among College Students. Alcohol. Res..

[6] Burns S., Jancey J., Crawford G., Hallett J., Portsmouth L., Longo J. (2016). A Cross Sectional Evaluation of an Alcohol Intervention Targeting Young University Students. BMC Public Health.

[7] Tanner-Smith E.E., Lipsey M.W. (2015). Brief Alcohol Interventions for Adolescents and Young Adults: A Systematic Review and Meta-Analysis. J. Subst. Abus. Treat..

[8] Grossbard J.R., Mastroleo N.R., Geisner I.M., Atkins D., Ray A.E., Kilmer J.R., Mallett K., Larimer M.E., Turrisi R. (2016). Drinking Norms, Readiness to Change, and Gender as Moderators of a Combined Alcohol Intervention for First-Year College Students. Addict. Behav..

[9] Bem S.L. (1994). The Lenses of Gender: Transforming the Debate on Sexual Inequality.

[10] Haines E.L., Deaux K., Lofaro N. (2016). The Times They Are A-Changing … or Are They Not? A Comparison of Gender Stereotypes, 1983–2014. Psychol. Women Q..

[11] Hosoda M., Stone D.L. (2000). Current Gender Stereotypes and Their Evaluative Content. Percept. Mot. Ski..

[12] Wechsler H., Dowdall G.W., Davenport A., Rimm E.B. (1995). A Gender-Specific Measure of Binge Drinking among College Students. Am. J. Public Health.

[13] Blank M.-L., Connor J., Gray A., Tustin K. (2015). Screening for Hazardous Alcohol Use among University Students Using Individual Questions from the Alcohol Use Disorders Identification Test-Consumption. Drug Alcohol. Rev..

[14] Wicki M., Kuntsche E., Gmel G. (2010). Drinking at European Universities? A Review of Students’ Alcohol Use. Addict. Behav..

[15] Dawson K.A., Schneider M.A., Fletcher P.C., Bryden P.J. (2007). Examining Gender Differences in the Health Behaviors of Canadian University Students. J. R. Soc. Promot. Health.

[16] Krieg D.B., Krause A.K. (2017). Drinking Like a Man: How Gender Norms Influence College Students’ Perceptions of Binge Drinkers. Discourses on Gender and Sexual Inequality.

[17] Lebreton F., Peralta R.L., Allen-Collinson J., Wiley L.C., Routier G. (2017). Contextualizing Students’ Alcohol Use Perceptions and Practices within French Culture: An Analysis of Gender and Drinking among Sport Science College Students. Sex Roles.

[18] Clinkinbeard S.S., Barnum T.C. (2017). Gendered Self-Concepts and Drinking Behavior in a National Sample of Emerging Adults. Fem. Criminol..

[19] Peralta R.L., Steele J.L., Nofziger S., Rickles M. (2010). The Impact of Gender on Binge Drinking Behavior Among U.S. College Students Attending a Midwestern University: An Analysis of Two Gender Measures. Fem. Criminol..

[20] Wells S., Flynn A., Tremblay P.F., Dumas T., Miller P., Graham K. (2014). Linking Masculinity to Negative Drinking Consequences: The Mediating Roles of Heavy Episodic Drinking and Alcohol Expectancies. J. Stud. Alcohol Drugs.

[21] Angosta J., Steers M.-L.N., Steers K., Lembo Riggs J., Neighbors C. (2019). Who Cares If College and Drinking Are Synonymous? Identification with Typical Students Moderates the Relationship between College Life Alcohol Salience and Drinking Outcomes. Addict. Behav..

[22] Bravo A.J., Prince M.A., Pearson M.R. (2017). College-Related Alcohol Beliefs and Problematic Alcohol Consumption: Alcohol Protective Behavioral Strategies as a Mediator. Subst. Use Misuse.

[23] Lui P.P. (2019). College Alcohol Beliefs: Measurement Invariance, Mean Differences, and Correlations with Alcohol Use Outcomes across Sociodemographic Groups. J. Couns. Psychol..

[24] DiBello A.M., Miller M.B., Carey K.B. (2019). Positive Heavy Drinking Attitude Mediates the Association between College Alcohol Beliefs and Alcohol-Related Outcomes. Addict. Behav..

[25] Pearson M.R. (2013). Use of Alcohol Protective Behavioral Strategies among College Students: A Critical Review. Clin. Psychol. Rev..

[26] Arterberry B.J., Smith A.E., Martens M.P., Cadigan J.M., Murphy J.G. (2014). Protective Behavioral Strategies, Social Norms, and Alcohol-Related Outcomes. Addict. Res. Theory.

[27] Clarke N., Kim S.-Y., Ray A.E., White H.R., Jiao Y., Mun E.-Y. (2016). The Association Between Protective Behavioral Strategies and Alcohol-Related Problems: An Examination of Race and Gender Differences among College Drinkers. J. Ethn. Subst. Abus..

[28] Martin R.J., Cremeens J.L., Umstattd M.R., Usdan S.L., Talbott-Forbes L., Garner M.M. (2012). Drinking Behaviour, Protective Behavioural Strategies and School Performance of College Students. Drugs Educ. Prev. Policy.

[29] Tabernero C., Gutiérrez-Domingo T., Luque B., García-Vázquez O., Cuadrado E. (2019). Protective Behavioral Strategies and Alcohol Consumption: The Moderating Role of Drinking-Group Gender Composition. Int. J. Environ. Res. Public. Health.

[30] Daigle L.E., Johnson L.M., Napper S.L., Azimi A.M. (2016). Protective Behavioural Strategies While Drinking: Do They Protect against Sexual Victimisation and Is This Protection Gendered?. Drug Alcohol. Rev..

[31] Labrie J.W., Lac A., Kenney S.R., Mirza T. (2011). Protective Behavioral Strategies Mediate the Effect of Drinking Motives on Alcohol Use among Heavy Drinking College Students: Gender and Race Differences. Addict. Behav..

[32] Whitley R.B., Madson M.B., Zeigler-Hill V. (2018). Protective Behavioral Strategies and Hazardous Alcohol Use among Male College Students: Conformity to Male Gender Norms as a Moderator. Psychol. Men. Masculinity.

[33] Reinert D.F., Allen J.P. (2007). The Alcohol Use Disorders Identification Test: An Update of Research Findings. Alcohol. Clin. Exp. Res..

[34] Babor T.F., Higgins-Biddle J.C., Saunders J.B., Monteiro M.G. (2001). The Alcohol Use Disorders Identification Test: Guidelines for Use in Primary Care.

[35] DeMartini K.S., Carey K.B. (2012). Optimizing the Use of the AUDIT for Alcohol Screening in College Students. Psychol. Assess.

[36] Fleming M.F., Barry K.L., Macdonald R. (1991). The Alcohol Use Disorders Identification Test (AUDIT) in a College Sample. Int. J. Addict..

[37] Kokotailo P.K., Egan J., Gangnon R., Brown D., Mundt M., Fleming M. (2004). Validity of the Alcohol Use Disorders Identification Test in College Students. Alcohol. Clin. Exp. Res..

[38] Bem S.L. (1974). The Measurement of Psychological Androgyny. J. Consult. Clin. Psychol..

[39] Fernández J.L.F., Coello M.T. (2010). Do the BSRI and PAQ Really Measure Masculinity and Femininity?. Span. J. Psychol..

[40] Osberg T., Atkins L., Buchholz L., Shirshova V., Swiantek A., Whitley J., Hartman S., Oquendo N. (2010). Development and Validation of the College Life Alcohol Salience Scale: A Measure of Beliefs About the Role of Alcohol in College Life. Psychol. Addict. Behav. J. Soc. Psychol. Addict. Behav..

[41] Martens M.P., Pederson E.R., Labrie J.W., Ferrier A.G., Cimini M.D. (2007). Measuring Alcohol-Related Protective Behavioral Strategies among College Students: Further Examination of the Protective Behavioral Strategies Scale. Psychol. Addict. Behav..

[42] Treloar H., Martens M.P., McCarthy D.M. (2015). The Protective Behavioral Strategies Scale–20: Improved Content Validity of the Serious Harm Reduction Subscale. Psychol. Assess.

[43] Prince M.A., Carey K.B., Maisto S.A. (2013). Protective Behavioral Strategies for Reducing Alcohol Involvement: A Review of the Methodological Issues. Addict. Behav..

[44] Martens M.P., Ferrier A.G., Sheehy M.J., Corbett K., Anderson D.A., Simmons A. (2005). Development of the Protective Behavioral Strategies Survey. J. Stud. Alcohol..

[45] RStudio Team (2023). RStudio: Integrated Development for R. RStudio.

[46] Lumley T. (2004). Analysis of Complex Survey Samples: R Package Verson 2.2. J. Stat. Softw..

[47] Ristori J., Cocchetti C., Romani A., Mazzoli F., Vignozzi L., Maggi M., Fisher A.D. (2020). Brain Sex Differences Related to Gender Identity Development: Genes or Hormones?. Int. J. Mol. Sci..

[48] Canadian Centre on Substance Abuse (2018). Heavy Episodic Drinking Among Post-Secondary Students: Influencing Factors and Implications.

[49] Carey K.B., Scott-Sheldon L.A.J., Carey M.P., DeMartini K.S. (2007). Individual-Level Interventions to Reduce College Student Drinking: A Meta-Analytic Review. Addict. Behav..

[50] Borsari B., Merrill J.E., Yurasek A., Miller M.B., Carey K.B. (2016). Does a Brief Motivational Intervention Reduce Frequency of Pregaming in Mandated Students?. Subst. Use Misuse.

[51] Barnett N.P., Orchowski L.M., Read J.P., Kahler C.W. (2013). Predictors and Consequences of Pregaming Using Day- and Week-Level Measurements. Psychol. Addict. Behav..

[52] Glindemann K.E., Ehrhart L.J., Maynard M.L., Geller E.S. (2006). Letter to the Editor: Alcohol Front-Loading Among College Students: Exploring the Need for Prevention Intervention. J. Alcohol. Drug Educ..

[53] Kypri K., Maclennan B., Connor J. (2020). Alcohol Harms over a Period of Alcohol Policy Reform: Surveys of New Zealand College Residents in 2004 and 2014. Int. J. Environ. Res. Public Health.

[54] Kypri K., Maclennan B., Cousins K., Connor J. (2018). Hazardous Drinking among Students over a Decade of University Policy Change: Controlled Before-and-After Evaluation. Int. J. Environ. Res. Public Health.

[55] Cousins K., Connor J.L., Kypri K. (2014). Effects of the Campus Watch Intervention on Alcohol Consumption and Related Harm in a University Population. Drug Alcohol. Depend..

[56] D’Amico E.J., Parast L., Shadel W.G., Meredith L.S., Seelam R., Stein B.D. (2018). Brief Motivational Interviewing Intervention to Reduce Alcohol and Marijuana Use for At-Risk Adolescents in Primary Care. J. Consult Clin. Psychol..

[57] Martín-Pérez C., Navas J.F., Perales J.C., López-Martín Á., Cordovilla-Guardia S., Portillo M., Maldonado A., Vilar-López R. (2019). Brief Group-Delivered Motivational Interviewing Is Equally Effective as Brief Group-Delivered Cognitive-Behavioral Therapy at Reducing Alcohol Use in Risky College Drinkers. PLoS ONE.

[58] McHugh R.K., Hearon B.A., Otto M.W. (2010). Cognitive-Behavioral Therapy for Substance Use Disorders. Psychiatr. Clin. N. Am..

[59] Hoffman R.M., Borders L.D. (2001). Twenty-Five Years After the Bem Sex-Role Inventory: A Reassessment and New Issues Regarding Classification Variability. Meas. Eval. Couns. Dev..

[60] Keener E. (2015). The Complexity of Gender: It Is All That and More….In Sum, It Is Complicated. Sex Roles.

[61] Choi N., Fuqua D.R. (2003). The Structure of the Bem Sex Role Inventory: A Summary Report of 23 Validation Studies. Educ. Psychol. Meas..

